# Determinants of pneumococcal vaccination behavior among elderly people aged ≥60 years in Jiangxi, China

**DOI:** 10.1097/MD.0000000000042597

**Published:** 2025-05-30

**Authors:** Han Zhao, Shicheng Guo, Jie Liu, Li Li, Jing Wu

**Affiliations:** a Immunization Program Institute, Jiangxi Provincial Key Laboratory of Major Epidemic Prevention and Control, Jiangxi Provincial Center for Disease Control and Prevention, Nanchang, Jiangxi Province, China; b School of Public Health, Jiangxi Medical College, Nanchang University, Nanchang, Jiangxi Province, China; c Jiangxi Provincial Key Laboratory of Disease Prevention and Public Health, Nanchang University, Nanchang, Jiangxi Province, China; d The Immunization Planning Center, Chinese Center for Disease Control and Prevention, Beijing, China.

**Keywords:** elderly people, influence factor, pneumococcal vaccine, vaccination behavior

## Abstract

The aim of this study was to explore factors influencing pneumococcal vaccination in people aged ≥ 60 years. We used a non-matched case-control study design to collect data on PPSV23 administration and pneumococcal disease/PPSV23 cognition and other factors among ≥ 60-year-olds in 2 prefectures of Jiangxi Province from January 2022 to December 2023. We compared the composition of each factor between vaccinated and non-vaccinated subjects, identified factors influencing pneumococcal vaccination using a logistic regression model and evaluated the model predictive ability by using ROC curve. This study included 212 subjects who received PPSV23 and 213 subjects who did not receive. The analysis of knowledge and attitude of pneumococcal disease/PPSV23 showed that the proportion of awareness ranged from 13.21% to 84.43% in the vaccinated group and from 13.62% to 67.14% in the unvaccinated group, and the proportion of positive attitudes ranged from 39.15% to 89.15% in the vaccinated group and 30.52% to 63.38% in the unvaccinated group. Multivariate stepwise logistic regression analysis showed that urban residents (aOR = 2.603, 95% CI: 1.236–5.481), PPSV23 vaccination history of surrounding people (aOR = 8.152, 95% CI: 4.832–13.751), healthcare workers recommended PPSV23 (aOR = 2.960, 95% CI: 1.763–4.968), acceptable vaccine price at ≥ 200 yuan (aOR = 3.061, 95% CI: 1.461–6.412), and positive attitude toward pneumococcal disease/PPSV23 (aOR = 4.636, 95% CI: 1.492–14.411) promoted PPSV23 vaccination of subjects. The pneumococcal vaccination of ≥ 60-year-olds in Jiangxi was mainly influenced by PPSV23 vaccination history of surrounding people, pneumococcal disease/PPSV23 cognition, and vaccine price. It is suggested that health education on knowledge about pneumococcal disease/PPSV23 be strengthened for the elderly.

## 1. Introduction

Pneumococci is the main cause of invasive pneumococcal disease (IPD), which can cause meningitis, septicemia and pneumonia. It is also the most common pathogen of adult community-acquired pneumonia (CAP).^[1]^ Pneumococcal pneumonia is one of the leading causes of morbidity and mortality in the elderly population in China,^[2]^ with 125,000 deaths due to pneumonia each year, and the prevalence of pneumococcal infection ranging from 28.0% to 71.5%.^[1,3]^ As the number of elderly people in China increases, the burden of disease due to pneumococcal disease increase subsequently. Sun et al reported that the incidence of CAP increased with age, ranging from 7.8/1000 person-years to 14.9/1000 person-years.^[4]^ The Chinese antimicrobial drug resistance detection system showed that pneumococcal resistance was as high as 40% in the elderly aged 65 years and older.^[5]^ Pneumococcal vaccination is an effective means of preventing pneumococcal pneumonia, and pneumococcal disease can be effectively controlled by reducing transmission and mitigating antibiotic resistance through vaccination.^[6]^ In China, 2 vaccines are currently approved for pneumonia protection, including the 13-valent pneumococcal polysaccharide conjugate vaccine (PCV13), which is indicated for infants and children between 6 weeks of age and 5 years of age, and the 23-valent pneumococcal polysaccharide vaccine (PPCV23), which is intended for use in people 2 years of age and older.^[7]^ According to the 2012 Updated Guidelines for the Prevention of Pneumococcal Diseases of the Chinese Society of Preventive Medicine, PPSV23 is indicated for the elderly population aged 60 years and older and for patients with chronic diseases.^[8]^ Several studies have demonstrated its efficacy in preventing pneumonia and IPD.^[6,9–12]^ In the following years of PPSV23 vaccination in the elderly, the rates of lower respiratory tract infections (69.7%) and hospitalizations (65.9%) declined.^[13]^

Currently, most developed countries have a pneumococcal vaccine coverage rate of more than 50% for older persons.^[14–19]^ Nevertheless, epidemiological surveillance data reveal substantial geographic disparities in PPSV23 vaccination coverage among older adults across China. According to relevant research studies, the vaccination rate of PPSV23 for the elderly exhibited markedly lower in Guangzhou, Ningbo and Changsha which ranged from 5.99% to 14.13%, and demonstrated markedly lower immunization coverage compared to municipalities implementing public-funded immunization initiatives for adults ≥ 60 years (42.09% in Chengdu, 37.60% in Shanghai, and 40.39% in Changshu).^[20–25]^ A previous survey on PPSV23 vaccination reported that PPSV23 vaccination rates varied by age, with substantial variability in vaccination coverage ranging from 12.14% to 53.94% for children under 14 years of age, contrasting sharply with ≤ 1% uptake observed in individuals over 15 years.^[26]^

Vaccination is an outcome behavior that results from a complex decision-making process that can be potentially influenced by a variety of factors, such as disease or vaccine perceptions and beliefs, financial barriers, medical care, and available health education resources Numerous studies have shown that both higher household economic status and adequate awareness levels regarding pneumonia vaccination serve as key determinants of PPSV23 vaccination behavior among older adults.^[17,19,20,27–31]^ Multiple investigations conducted in China indicated that limited understanding of pneumococcal disease and its vaccines, skepticism regarding vaccine efficacy, and prevalent safety-related apprehensions including contraindication considerations were key barriers to the low vaccination rate of PPSV23.^[21,32–35]^ Prior epidemiological evidence from Japan reported that poorer access to health care is associated with lower likelihood of vaccination.^[16,36]^ In addition, a multi-provincial population-based analysis across China revealed that healthcare worker recommendations positively influenced PPSV23 vaccination behavior in older adults.^[37]^ Understanding the factors related to vaccination in the elderly is extremely important to improve the vaccination rate, and there are few research studies investigating the potential barriers to PPSV23 vaccination among the elderly in China. Research into PPSV23 vaccination and their determining factors among older adults were not widely available, and a deeper understanding of influencing factors is needed if vaccine rates are to improve. This study aimed to understand pneumococcal diseases and their vaccine perceptions and PPSV23 vaccination behaviors of the elderly aged 60 years and older in Jiangxi Province, and to explore the influencing factors of PPSV23 vaccination behavior, which will provide a reference basis to improve the vaccination rate.

## 2. Materials and methods

### 2.1. Subjects

This study utilized a non-matched case-control study design, in which older adults ≥ 60 years were surveyed and divided into a PPSV23-vaccinated group (“case” group) and a PPSV23-unvaccinated group (“control” group). Inclusion criteria for the vaccinated group: Date of birth no later than September 30, 1963, based on ID card; actual place of residence for more than 6 months at the start of the survey; vaccinated with PPSV23 from January 1, 2022, to the date of the survey, based on the vaccination record of the Jiangxi Provincial Immunization Planning Information System; Informed consent and voluntary participation in the survey. Inclusion criteria for the unvaccinated group: date of birth no later than September 30, 1963, based on the identity card; actual place of residence for more than 6 months at the start of the survey; not vaccinated with PPSV23 as the day of the survey, based on the vaccination record of the Jiangxi Provincial Immunization Planning Information System; informed consent and voluntary participation in the survey. Exclusion criteria: respondent has a communication disability (speech impediment, deafness, severe mental illness or cognitive impairment); individuals who do not have a guardian/proxy to assist in completing the survey; Failure to provide written informed consent; Participants aged < 60 years were excluded from the study.

### 2.2. Methods

#### 2.2.1. Sample size calculation

According to the sample size calculation formula for unmatched case–control studies, $n_{1}={\left(z_{\alpha }+z_{\beta }\right)}^{2}\times (1+1/r)\times \overset{–}{p}\overset{–}{q}/{\left(p_{1}-p_{0}\right)}^{2},$ $\overset{–}{p}=(p_{1}+rp_{0})/(1+r),$ $p_{1}=p_{0}RR/[1+p_{0}(RR-1)]$, the required sample size was calculated, n_1_ was the sample size of the vaccination group, *p*_0_ was the probability of a factor in the unvaccinated group, and *p*_1_ was the probability of a factor in the vaccinated group, and the OR value was used as an approximation of RR, and it was obtained by reviewing the literature^[21,22,24,38–40]^ that *p*_0_ = 0.480, OR = 2. Given the significance level α = 0.05, and the degree of certainty (1 − β)= 0.9, the ratio of sample size between vaccinated and unvaccinated group was 1:1 (*R* = 1), considering the 10% nonresponse rate and the maneuverability of the specific distribution of samples in the field, it was calculated that the sample size of the vaccinated group and unvaccinated group were both 216, respectively.

#### 2.2.2. Data collection

Multi-stage stratified cluster sampling was used to randomly select 2 prefecture-level cities in Jiangxi Province, with 1 urban area and 1 county in each city. In this study, Honggutan District and Nanchang County, Zhanggong District and Yudu County were selected. A total of 108 participants were included (54 vaccinated and 54 unvaccinated) from each surveyed county/district. Of these, 30 cases were 60 to 64 years old, 30 cases were 65 to 69 years old, 24 cases were 70 to 74 years old, 12 cases were 75 to 79 years old, and 12 cases were ≥ 80 years old. It is also required that the number of people surveyed in the vaccinated and unvaccinated groups is basically the same in terms of age, and that the ratio of male to female survey respondents in each age group is between 1:1 and 1:1.5. Vaccinated group sampling through the vaccination unit information system to retrieve the list of results that meet the inclusion criteria of the vaccination group, according to the age of 60 to 64 years old, 65 to 69 years old, 70 to 74 years old, 75 to 79 years old, ≥80 years old is divided into 5 age groups, each group in accordance with the systematic sampling method of sampling the number of cases of the corresponding sample size, and ultimately get the list of respondents to the survey of the vaccinated group. Sampling of the unvaccinated group was conducted based on elderly health examination records, universal health records, and related information from streets/townships where vaccination units were located. These records were cross-checked with vaccination records in the immunization information system to obtain a list of eligible unvaccinated individuals meeting the inclusion criteria. The final sample was stratified into 5 age groups (60–64, 65–69, 70–74, 75–79, and ≥80 years), with systematic sampling applied to select the predetermined sample size from each group, ultimately yielding the study participants for the unvaccinated group.

#### 2.2.3. Questionnaire survey

This questionnaire was adapted from WHO’s behavioral and social drivers (BeSD) tools^[41]^ of vaccination with 4 domains: thinking and feeling, social processes, motivation, and practical issues, which influence the decision-making process during vaccination-related behavior. We extracted influencing factors and evaluating content validity through literature search and multiple rounds of expert consultation, as well as conducting a questionnaire and multiple rounds of cognitive interview testing to determine the final entries to be used in the questionnaire. The questionnaire was administered to respondents in December 2023 using a face-to-face survey consisting of 7 sections. The first part focused on assessing the socio-demographic characteristics of the respondents, including age, sex, marital status, education level, residence, number of household members (number of people living together for > 6 months, including themselves) and annual household income. The second component was health status, including history of chronic diseases (including hypertension, diabetes, cardiovascular and cerebrovascular diseases, chronic obstructive pulmonary diseases, other respiratory diseases, malignant tumors, renal diseases, liver diseases, and other chronic noncommunicable diseases), influenza-like symptoms, and physical health status. The third part was knowledge about pneumococcal diseases and PPSV23, which assessed respondents’ knowledge of pneumococcus, pneumococcal diseases, and pneumococcal vaccine. The fourth part was to assess the respondents’ attitudes towards pneumococcal diseases and PPSV23. The fifth part is the influence of the surrounding people (including family members, relatives, and friends), including the history of pneumococcal diseases and pneumococcal vaccination of the surrounding people, as well as the recommendations of healthcare workers. The sixth part is vaccination services, including the price of pneumococcal vaccine. The seventh part was vaccination-related factors, including access to PPSV23 vaccination information and reasons for PPSV23 vaccination and non-vaccination. The questionnaire is included in the Supplementary Materials, Supplemental Digital Content, https://links.lww.com/MD/P37.

#### 2.2.4. Relevant definitions and standards

Variable assignment: Pneumococcal diseases and PPSV23 related knowledge questions section, including 10 questions, out of 10 points, each question choose “yes” counts as 1 point (considered “knowledgeable”), choose “no” or “don’t know” counts as 0 points (considered “not knowledgeable”). The knowledge factor independent variable was converted to a dichotomous variable, and a score of >60% of the total score for the 10 questions was considered to be good knowledge, and the opposite was considered to be poor knowledge; Pneumococcal diseases and PPSV23 attitudinal questions section, including 6 questions, out of 30 points. For each question, option 1 scores 5 points, option 2 scores 4 points, option 3 scores 3 points, option 4 scores 2 points, and option 5 scores 1 point, options 1 and 2 are considered as positive attitude and options 3, 4 and 5 as negative attitude. The attitude factor independent variable was converted to a dichotomous variable, and a score of >60% of the total score for the 6 questions was considered to be positive attitude, and the opposite was considered to be negative attitude.^[42,43]^

### 2.3. Quality control

Before the survey, the investigators were trained to standardize the questioning methods and survey techniques for both the vaccinated and unvaccinated groups. During the survey, the questionnaire was completed by the investigator through one-on-one interviews, and each question was explained in detail, and some survey techniques were used to help respondents recall associations, so that the respondents could answer more accurately; at the same time, on-site audits were conducted on the survey data, and if any missing items or logical errors were found, timely verifications with the respondents were made to supplement and correct the problems. After the survey, all questionnaires were double-entered independently using Epidata 3.1, followed by data consistency checks to ensure completeness and accuracy.

### 2.4. Statistical analysis

SPSS26.0 software was used to organize and analyze the data. Reliability and validity tests were conducted for the Pneumococcal Severe Disease and PPSV23 Attitude Scale. Cronbach alpha coefficient was used to assess the internal consistency reliability, when the reliability coefficient ≥0.8 indicated excellent, ≥0.7 indicated good, and ≥0.6 indicated acceptable. Exploratory factor analysis was used to reflect the structural validity of the scale, and the Kaiser–Meyer–Olkin (KMO) test and Bartlett Test of Spherical were used to assess whether the data were suitable for factor analysis, and the KMO value > 0.70 and Bartlett test of spherical *P* < .05 indicated that there were common factors in the scale and it was suitable for factor analysis. The extraction method of exploratory factor analysis was principal component analysis (PCA), and the rotation method was maximum variance orthogonal rotation, and the entries with factor loading values < 0.4 after orthogonal rotation were deleted, and the cumulative variance contribution rate ≥ 40%, indicating acceptable structural validity. The factors were analyzed descriptively using frequency counts and composition ratios; the crude odds ratios (OR) and their 95% confidence intervals (CI) of the factors in the 2 groups were calculated using univariate logistic regression model; and in order to adjust for the effects of confounding factors, the factors that were significant in the univariate analysis were progressively included in the multivariate stepwise logistic regression model (α_in_ = 0.05, α_out_ = 0.1) to analyze the final influencing factors of PPSV23 vaccination, and calculate the adjusted OR and its 95% CI. The R4.3.1 software was used to analyze the model’s area under curve (AUC) for the receiver operating characteristic to evaluate the model. The test level was taken as α = 0.05 (2-sided).

### 2.5. Ethical consideration

This study was ethically reviewed by the Ethics Committee of the Chinese Center for Disease Control and Prevention (Grant No. 202325), and all respondents signed an informed consent form.

## 3. Results

### 3.1. Basic demographic characteristics of the subjects

A total of 432 questionnaires were distributed and 432 questionnaires (100%) were returned, of which 425 (98.38%) were valid. Among the 425 survey respondents, 49.88% (212) were vaccinated PPSV23 and 50.12% (213) unvaccinated PPSV23. 48.71% (207) were male and 51.29% (218) female. 28.00% (119) were 60 to 64 years old, 27.29% (116) were 65 to 69 years old, 22.29% (116) were 70 to 74 years old, 11.29% (48) were 75 to 79 years old, and 10.82% (46) were ≥ 80 years old. Married and divorced/widowed accounted for 88.00% (374) and 12.00% (51), respectively. Elementary school and below, junior high school, middle school/high school and above accounted for 49.65% (211), 25.88% (110), 24.47% (104), respectively. Urban and rural residence accounted for 85.65% (364) and 14.35% (61), respectively. The distribution of family size was 36.47% (155) for 1 to 2 persons, 26.12% (111) for 3 to 4 persons, and 37.41% (159) for ≥ 5 persons. The annual income of the household was 23.76% (101) for 0 to 40,000 yuan, 101) for 0 to 40,000 yuan, and 12.00% (51) were 0 to 40,000 yuan, 35.76% (152) were 50 to 90,000 yuan, and 40.47% (172) were ≥ 100,000 yuan.

### 3.2. Knowledge of pneumococcal disease/PPSV23 in older adults

The proportion of awareness of the respondents in the unvaccinated and vaccinated groups on the 10 knowledge questions on pneumococcal diseases/PPSV23 ranged from 13.62% to 67.14% and 13.21% to 84.43%, respectively. In the unvaccinated group, the question with the highest proportion of awareness was “Pneumococcus can be spread in the air when an infected person coughs or sneezes” (67.14%), and in the vaccinated group it was “Elderly people ≥ 60 years of age are the preferred group to be vaccinated against PPSV23” (84.43%). The question “Pneumococcal infection can cause meningitis” was least correctly answered by the unvaccinated and vaccinated groups, with proportion of awareness of 13.62% and 13.21%, respectively (Table 1).

**Table 1:** Subject’ answers to knowledge questions regarding pneumococcal disease/PPSV23 vaccine.

Question	Options	Unvaccinated group (n = 213)	Vaccinated group (n = 212)
		Frequency (%)	Frequency (%)
Pneumococcus bacteria can be spread in the air when an infected person coughs or sneezes	Yes	143 (67.14)	169 (79.72)
	No	6 (2.82)	4 (1.89)
	Don’t know	64 (30.05)	39 (18.40)
Pneumococcal infection can cause otitis media	Yes	86 (40.38)	58 (27.36)
	No	15 (7.04)	21 (9.96)
	Don’t know	112 (52.58)	133 (62.74)
Pneumococcal infection can cause meningitis	Yes	29 (13.62)	28 (13.21)
	No	64 (30.05)	41 (19.34)
	Don’t know	120 (56.34)	143 (67.45)
Vaccination can prevent pneumococcal diseases	Yes	115 (53.99)	160 (75.47)
	No	17 (7.98)	3 (1.42)
	Don’t know	81 (38.03)	49 (23.11)
Fever may occur after PPSV23 vaccination	Yes	69 (32.39)	94 (44.34)
	No	9 (4.23)	38 (17.92)
	Don’t know	135 (63.38)	80 (37.74)
People over 60 yr old are the priority groups for PPSV23 vaccination	Yes	123 (57.75)	179 (84.43)
	No	10 (4.69)	6 (2.83)
	Don’t know	80 (37.56)	27 (12.74)
Patients with cardiovascular disease are the priority group for PPSV23 vaccination	Yes	96 (45.07)	153 (72.17)
	No	17 (7.98)	12 (5.66)
	Don’t know	100 (46.95)	47 (22.17)
People with low immunity are the priority groups for PPSV23 vaccination	Yes	118 (55.40)	176 (83.02)
	No	9 (4.23)	4 (1.89)
	Don’t know	86 (40.38)	32 (15.09)
Hypersensitivity to any PPSV23 component is a contraindication to vaccination in the elderly	Yes	100 (46.95)	102 (48.11)
	No	4 (1.88)	7 (3.30)
	Don’t know	109 (51.17)	103 (48.58)
Chronic diseases with unstable control are contraindications to vaccination in the elderly	Yes	50 (23.47)	55 (25.94)
	No	42 (19.72)	27 (12.74)
	Don’t know	121 (56.81)	130 (61.32)

PPSV23 = 23-valent pneumococcal polysaccharide vaccine.

### 3.3. Pneumococcal pneumococcal disease/PPSV23 attitudinal awareness in older adults

The KMO = 0.813 and Bartlett Test of Spherical *P* < 0. 001 in this study indicated that the data were suitable for factor analysis. The results of factor analysis showed that PCA produced 2 factors with Cronbach α values of 0.678 and 0.890, and the factor loadings of all the observed variables were in the range of 0.579 to 0.914, which were all >0.4, indicating that the items of the scale were set up reasonably well, and the cumulative variance contribution of the 2 factors was 74.69% (Table 2). The proportion of positive attitudes of the survey respondents in the unvaccinated group and the vaccinated group for the 6-item pneumococcal disease/PPSV23 attitude questions ranged from 30.52% to 63.38% and 39.15% to 89.15%, respectively. The highest percentages of positive attitudes were found in the unvaccinated and vaccinated groups for the “very safe/comparatively safe” option of “PPSV23 safety,” with positive attitudes held by 63.38% and 89.15%, respectively. The lowest percentage was found on the “very high/higher” option for “risk of pneumococcal infection,” with positive attitudes of 30.52% and 39.15%, respectively (Table 3).

**Table 2:** Factor analysis findings on attitudes towards pneumococcal disease/PPSV23.

Factor	Loadings	variance contribution rate (%)	Cumulative variance contribution rate (%)	Cronbach alpha
1		46.35	46.35	0.678
PPSV23 validity	0.914			
PPSV23 security	0.888			
Importance of PPSV23 vaccination for health	0.847			
2		28.34	74.69	0.890
Risk of pneumococcal infection	0.853			
Pneumococcal infectivity	0.742			
Degree of health damage caused by pneumococci	0.579			

PPSV23 = 23-valent pneumococcal polysaccharide vaccine.

**Table 3:** Subject’ responses to items assessing attitudes towards pneumococcal disease/PPSV23.

Question	Options	Unvaccinated group (n = 213)	Vaccinated group (n = 212)
		Frequency (%)	Frequency (%)
Risk of pneumococcal infection	Very high	6 (2.82)	12 (5.66)
	Fairly high	59 (27.70)	71 (33.49)
	Normal	104 (48.83)	108 (50.94)
	Low	22 (10.33)	21 (9.96)
	Very low	22 (10.33)	0 (0)
Pneumococcal infectivity	Very high	13 (6.10)	15 (7.08)
	Fairly high	83 (38.97)	88 (41.51)
	Normal	95 (44.60)	95 (44.81)
	Low	16 (7.51)	12 (5.66)
	Very low	6 (2.82)	2 (0.94)
Degree of health damage caused by pneumococci	Very serious	8 (3.76)	35 (16.51)
	Fairly serious	114 (53.52)	135 (63.68)
	Normal	80 (37.56)	40 (18.87)
	Not too serious	10 (4.69)	2 (0.94)
	Not at all serious	1 (0.47)	0 (0)
Importance of PPSV23 vaccination for health	Very important	22 (10.33)	48 (22.64)
	Fairly important	97 (45.54)	118 (55.66)
	Normal	83 (38.97)	46 (21.70)
	Not too important	10 (4.69)	0 (0)
	Completely unimportant	1 (0.47)	0 (0)
PPSV23 validity	Very valid	23 (10.80)	40 (18.87)
	Fairly valid	107 (50.23)	145 (68.40)
	Normal	78 (36.62)	27 (12.74)
	Not too valid	5 (2.35)	0 (0)
	Completely invalid	0 (0)	0 (0)
PPSV23 security	Very safety	26 (12.21)	59 (27.83)
	Fairly safety	109 (51.17)	130 (61.32)
	Normal	76 (35.68)	21 (9.91)
	Not too safety	2 (0.94)	2 (0.94)
	Very unsafe	0 (0)	0 (0)

PPSV23 = 23-valent pneumococcal polysaccharide vaccine.

### 3.4. Analysis of factors influencing PPSV23 vaccination behavior in elderly people aged ≥ 60 years

**Figure 1.**
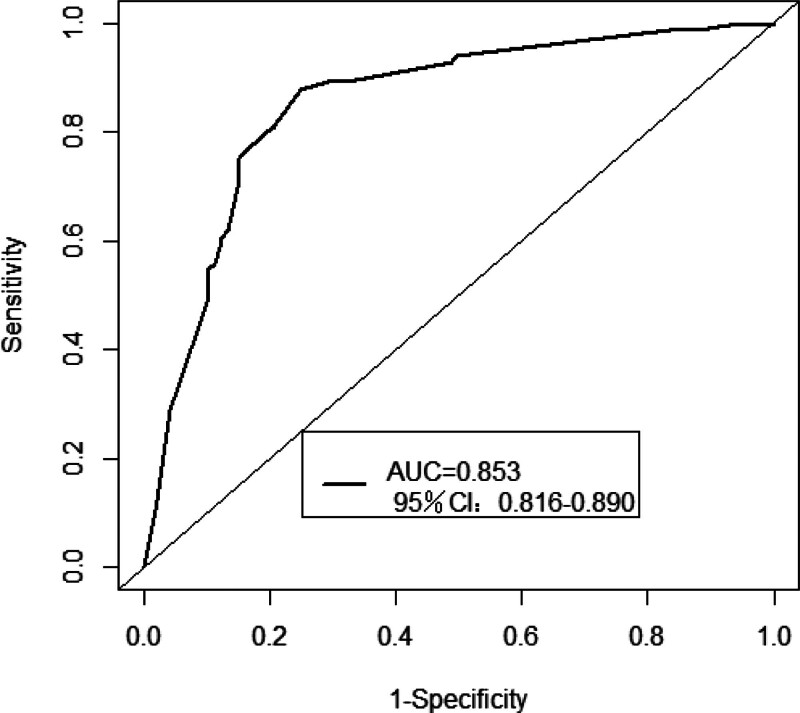
Stepwise logistic regression model ROC curve. The figure illustrates the ROC curve for the multivariate stepwise logistic regression model used in the study. The x-axis represents specificity, while the y-axis indicates 1-sensitivity. The AUC is a measure of the model’s predictive performance, with higher values indicating better discriminative ability. AUC = area under the curve, CI = confidence interval.

Univariate logistic regression analysis showed that residence, pneumococcal disease history of surrounding people, PPSV23 vaccination history of surrounding people, whether PPSV23 was recommended by healthcare professionals, acceptable vaccine price, knowledge of pneumococcal disease/PPSV23, and perceived attitudes toward pneumococcal disease/PPSV23 may be the factors influencing survey respondents’ PPSV23 vaccination behavior (Table 4). Multivariate stepwise logistic regression analysis showed that urban residents, PPSV23 vaccination history of surrounding people, healthcare workers recommended PPSV23, acceptable vaccine price at ≥ 200 yuan, and positive attitude toward pneumococcal disease/PPSV23 were the facilitators of the survey respondents’ PPSV23 vaccination (Table 5). The stepwise logistic regression model AUC was 0.853 (95% CI: 0.816–0.890), indicating a good identification ability of the model (Fig. 1).

**Table 4:** Univariate analysis of affecting PPSV23 vaccination behavior of elderly people aged ≥ 60 yr in Jiangxi Province.

Characteristic	Vaccinated group (n = 213)	Unvaccinated group (n = 212)	OR (95% CI)
	Frequency (%)	Frequency (%)	
Age (yr)			
60 to 64	60 (28.30)	59 (27.70)	Ref.
65 to 69	58 (27.36)	58 (27.23)	0.98 (0.59–1.64)
70 to 74	48 (22.64)	48 (22.54)	0.98 (0.57–1.68)
75 to 79	24 (11.32)	24 (11.27)	0.98 (0.50–1.92)
≥80	22 (11.38)	24 (11.27)	0.90 (0.46–1.78)
Sex			
Male	107 (50.47)	100 (46.95)	Ref.
Female	105 (49.53)	113 (53.05)	0.87 (0.59–1.27)
Marital status			
Married	185 (87.26)	189 (88.73)	Ref.
Divorced/widowed	27 (12.74)	24 (11.27)	1.15 (0.64–2.07)
Educational level			
Primary and below	111 (52.36)	100 (46.95)	Ref.
Junior high school	47 (22.17)	63 (29.58)	0.67 (0.42–1.07)
Secondary/high school and above	54 (25.47)	50 (23.47)	0.97 (0.61–1.56)
Place of residence			
Countryside	20 (9.43)	41 (19.25)	Ref.
Town	192 (90.57)	172 (80.75)	2.29 (1.29–4.06)**
Family size			
1 to 2	78 (36.79)	77 (36.15)	Ref.
3 to 4	55 (25.94)	56 (26.29)	0.97 (0.60–1.58)
≥5	79 (37.26)	80 (37.56)	0.98 (0.63–1.52)
Annual family income (10,000 yuan)			
0 to 4	50 (23.58)	51 (23.94)	Ref.
5 to 9	66 (31.13)	86 (40.38)	0.78 (0.47–1.30)
≥10	96 (45.28)	76 (35.68)	1.29 (0.79–2.11)
Chronic disease history			
No	115 (54.25)	112 (52.58)	Ref.
Yes	97 (45.75)	101 (47.42)	0.94 (0.64–1.37)
Flu-like symptoms like fever and cough			
No	63 (29.72)	64 (30.05)	Ref.
Yes	149 (70.28)	149 (69.95)	1.02 (0.67–1.54)
Condition			
Good	105 (49.53)	101 (47.42)	Ref.
Fair/poor	107 (50.47)	112 (52.58)	0.92 (0.63–1.35)
Heard negative things about the vaccine			
No	172 (18.87)	172 (19.25)	Ref.
Yes	40 (81.13)	41 (80.75)	0.98 (0.60–1.58)
History of pneumococcal disease in the surrounding crowd			
No	137 (64.62)	172 (80.75)	Ref.
Yes	75 (35.38)	41 (19.25)	2.30 (1.48–3.57)***
History of PPSV23 vaccination in the surrounding crowd			
No	55 (25.94)	174 (81.69)	Ref.
Yes	157 (74.06)	39 (18.31)	12.74 (8.01–20.25)***
Pneumococcal vaccine recommended by medical staff			
No	66 (31.13)	159 (74.65)	Ref.
Yes	146 (68.87)	54 (25.35)	6.51 (4.26–9.95)***
Acceptable vaccine price (yuan)			
<100	92 (43.40)	129 (60.56)	Ref.
100 to 200	70 (33.02)	66 (30.99)	1.49 (0.97–2.29)
≥200	50 (23.58)	18 (8.45)	3.90 (2.13–7.11)***
Knowledge of pneumococcal severe disease/PPSV23			
Poor	99 (46.70)	129 (60.56)	Ref.
Good	113 (53.30)	84 (39.44)	1.75 (1.19–2.58)**
Pneumococcal disease/PPSV23 attitudinal awareness			
Negative	6 (2.83)	189 (88.73)	Ref.
Positive	206 (97.17)	24 (11.27)	4.36 (1.74–10.90)**

PPSV23 = 23-valent pneumococcal polysaccharide vaccine, Ref.=reference, OR = odds ratio, CI = confidence interval.

** *P*＜0.01.

*** *P*＜0.001.

**Table 5:** Multivariate stepwise logistic regression analysis of influencing PPSV23 vaccination behaviors of elderly people aged ≥ 60 in Jiangxi Province.

Variables	β	SE	Wald	*P*	aOR (95% CI)
Residence (Ref.:Countryside)					
Town	0.957	0.380	6.340	.012	2.603 (1.236–5.481)
History of pneumococcal disease in the surrounding crowd (Ref.:No)					
Yes	2.098	0.267	61.849	<.001	8.152 (4.832–13.751)
History of PPSV23 vaccination in the surrounding crowd (Ref.:No)					
Yes	1.085	0.264	16.858	<.001	2.960 (1.763–4.968)
Acceptable vaccine price (Ref.:<100)					
100 to 199 yuan	0.207	0.274	0.572	.450	1.231 (0.719–2.107)
≥200 yuan	1.119	0.377	8.791	.003	3.061 (1.461–6.412)
Pneumococcal disease/PPSV23 attitudinal awareness (Ref.:Negative)					
Positive	1.534	0.579	7.027	.008	4.636 (1.492–14.411)

aOR = adjusted odds ratio, CI = confidence interval, PPSV23 = 23-valent pneumococcal polysaccharide vaccine, SE = standard error, Ref. = reference.

### 3.5. *Analysis of vaccine information acquisition channels and reasons for not vaccinated with* PPSV23

**Figure 2.**
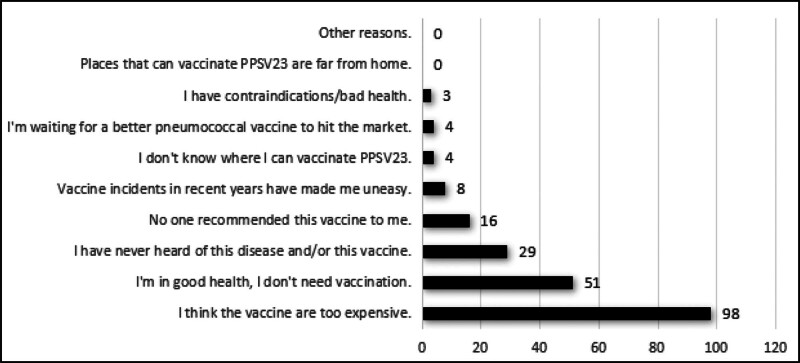
Top reasons for not vaccinated with PPSV23. PPSV23 = 23-valent pneumococcal polysaccharide vaccine.

Among the survey respondents in the vaccination group, being informed by family members or relatives, being publicized by vaccinating medical personnel, and being publicized on the Internet were the main channels for obtaining information about PPSV23 vaccination, accounting for 67.45% (143), 46.23% (98), and 14.62% (31), respectively. Among the survey respondents in the unvaccinated group, the price of the vaccine was too high, they were in good health and did not intend to get it, and they had not heard of this disease or vaccine were the main reasons for not being vaccinated against PPSV23, which accounted for 46.01% (98), 23.94% (51), and 13.62% (29), respectively (Fig. 2).

## 4. Discussion

Our study showed that the vaccination rate of the pneumococcal vaccine was 49.88%. Although the vaccination rate was increased compared with previous reports, almost half of older adults had not yet received vaccination. The results of the current study also revealed that older adults whose place of residence was rural were less likely to receive pneumococcal vaccine than that of lived in towns. The results of Shao et al showed that the willingness to vaccinate of rural residents was lower than that of urban residents, which is consistent with the results of our study, which may be related to the disparity in the distribution of income and health resources between urban and rural areas in China, as well as the inequality in the level of public health and medical care services.^[40]^ Consideration should be given to improving the accessibility and equity of vaccination services in rural areas in order to increase PPSV23 vaccination rates in this population. In this study, The proportion of people around (family, relatives and friends) who had been vaccinated with PPSV23 was 74.06% in the vaccinated group, while it was only 18.31% in the unvaccinated group. A study in Hong Kong showed that peers had a significant effect on improving confidence in pneumococcal vaccination among older adults.^[44]^ Therefore, utilizing the health transmission effect of parents of vaccinated children and peers of older adults to increase the chances of receiving PPSV23 vaccination by targeting the surrounding populations such as family members, relatives, and friends. Moreover, it might be a potentially promising strategy to increase the PPSV23 vaccination rate.^[40]^ According to a survey conducted in a Chinese population, physician’s recommendation (corrected OR = 7.40, 95% CI: 3.47–15.76, *P* = .008) was one of the main factors for older adults to receive pneumococcal vaccine.^[45]^ Similarly, a study in Japan also found that family doctor’s advice and health knowledge were associated with vaccination.^[19,29]^ This is similar to the results of the present study, in which a greater proportion of healthcare workers had recommended PPSV23 in the vaccination group, which may be explained by the fact that the advice of healthcare workers is a more reliable source of information; in addition, our study revealed the importance of advice from medical personnel; Public health workers and general practitioners (GPs) who were in direct contact with their patients could disseminate health-related knowledge and promote healthy behaviors among their patients. A study on patients’ behavioral changes found that generalization of health education by public health providers and GPs positively affected public health behaviors (e.g., vaccination).^[46–48]^ The majority of relevant studies have reported the importance of healthcare professionals recommending the expansion of pneumococcal vaccination coverage in target populations.^[19,49]^ In addition, public health workers are more likely to recommend PPSV23 vaccination than GPs, and promoting GP recommendations may be key to increasing vaccination coverage.^[37]^ Therefore, GPs should be provided with targeted health education training focusing on the interpretation of guidelines for the prevention of pneumococcal disease and the benefits of vaccination to encourage effective health promotion practices. Currently, PPSV23 is not included in the National Immunization Program in China and is an out-of-pocket expense in most areas.^[50]^ This study showed that older adults with an acceptable vaccine price of ≥ 200 RMB were more likely to receive PPSV23. A questionnaire survey conducted in China reported that price sensitivity played a key role in receiving PPSV23 vaccination among older adults (50–69 years old) in 13 communities. Several Chinese surveys reported that older adults with good family financial status could accept higher price vaccine costs and their vaccination rates were relatively high, while it was difficult for older adults with poor financial status to afford high vaccine costs.^[27,28]^ Therefore, measures such as government subsidies, adjustment of vaccine pricing, or inclusion of PPSV23 in health insurance are recommended to alleviate the financial barriers to self-payment for the vaccine. Vaccination willingness in older adults is related to the level of attitudinal cognition toward the vaccine and the disease it prevents.^[51]^ In this study, we found that older adults in the vaccination group had a better level of awareness of PPSV23, indicating that having a positive attitude toward the disease and the vaccine is a facilitator of vaccination behavior, suggesting that the vaccination rate of pneumonia vaccine among older adults can be improved by strengthening health education on the level of awareness of the attitude toward the disease and the vaccine.

Reasons for not vaccinating against PPSV23 showed that the price of the vaccine was too high, they were in good health and did not intend to get it; And they had not heard of the disease or the vaccine were the main reasons for not vaccinating against PPSV23. Several studies found that contraindications to vaccination, no need for vaccination, ill health, and the high cost of vaccination were the reasons for the reluctance to receive pneumococcal vaccine in the elderly population.^[32–34]^ The results of this study showed that there was no significant correlation between literacy level, history of chronic diseases, history of pneumonia and pneumococcal vaccination, which is consistent with the findings of Shao et al,^[32,40]^ and the possible explanation for this was that people with higher literacy levels were more likely to be skeptical about the safety and efficacy of the vaccine,^[52]^ and more research is needed to explore the reasons for the low level of vaccination in this population.

There are some limitations to this study. The respondents selected for this study are only a sample of the source population, and there are still some differences in some characteristics between the selected respondents and the unselected, making it difficult to avoid selection bias. In addition, the study could not directly estimate the causal relationship between the influencing factors and vaccination behavior, so its temporal sequence was difficult to judge. Despite these shortcomings, this study selected a large sample size and random sampling method, and took relevant quality control measures before, during, and after the survey, such as uniform training for investigators, one-on-one interviews, an on-site review system for survey information, 2-person back-to-back data entry and data consistency tests, and multivariate analysis, which reduced information bias and confounding bias. In addition, vaccination status was obtained from the Jiangxi Provincial Immunization Planning Information System, so this information is unlikely to be biased. We identified some variables independently associated with pneumococcal vaccination, which may help to improve vaccination strategies for the elderly.

## 5. Conclusions

The results of the analysis in this study suggest that residence, PPSV23 vaccination history of surrounding population, recommendation of healthcare professionals to recommend PPSV23, acceptable vaccine price and perceived attitudes toward pneumococcal disease/PPSV23 are associated with PPSV23 vaccination behavior among the elderly. Therefore, it is recommended that health education of the target population and healthcare workers about pneumococcal disease prevention be strengthened, as well as measures to reduce the cost of vaccination, in order to promote the vaccination of older adults with PPSV23.

## Acknowledgments

We would like to thank Nanchang and Ganzhou Municipal Centers for Disease Control and Prevention; all participating community health centers; and community neighborhood committees for their contributions to this study.

## Author contributions

**Conceptualization:** Jie Liu, Li Li.

**Data curation:** Han Zhao.

**Formal analysis:** Han Zhao.

**Investigation:** Shicheng Guo, Jing Wu.

**Writing – original draft:** Han Zhao.

**Writing – review & editing:** Jie Liu.

## Supplementary Material


