# Supplementary material for: Determinants of pneumococcal vaccination behavior among elderly people aged ≥60 years in Jiangxi, China
Source: Medicine (Baltimore). 2025 May 30;104(22):e42597. doi: 10.1097/MD.0000000000042597 (PMC12129538; doi:10.1097/MD.0000000000042597)
Supplement: Supplementary file 1 [file medi-104-e42597-s001.pdf]

Supplemental Digital Content1: Questionnaire on Factors Influencing Vaccination of the Elderly with PPSV23

Vaccinated Group Questionnaire:

Part1．Basic Information

1.Date of birth？（**Not later than 30 September 1963**）

□□□□year□□month□□date

2.Gender？

□Man □Women

3.Marital status？

□Married □Divorced/widowed

4.Educational level？

□Primary and below □Junior high school □Secondary/high school and above

5.Where have you lived most often in the past year？

□Countryside □Town

6.How many members of your family have lived together in the past year？

（**Note: Means living together for more than 6 months**）

□1-2 □3-4 □≥5

7.What was your annual household income in the past year?(10,000 yuan) (Note: Refers to the total annual income of household members living together)

□0-4 □5-9 □≥10

Part2．Health status

8.What doctor-diagnosed chronic illnesses have you had in the past year？

□No □Yes

9.In the past year, have you experienced flu-like symptoms such as fever and coughing？

①No ②Yes

10.How do you feel about your health over the past year？

□Good □Fair □Poor

Part3．Pneumococcal diseases and PPSV23 related knowledge

11.Do you think the following statements are correct？

（1）Pneumococcus bacteria can be spread in the air when an infected person coughs or sneezes.

①Yes ②No ③Don’t know

（2）Pneumococcal infection can cause otitis media.

①Yes ②No ③Don’t know

（4）Pneumococcal infection can cause meningitis.

①Yes ②No ③Don’t know

（4）Vaccination can prevent pneumococcal diseases.

①Yes ②No ③Don’t know

（5）Fever may occur after PPSV23 vaccination.

①Yes ②No ③Don’t know

12. Do you consider the following to be priority populations for PPSV23 vaccination?？

（1）People over 60 years old

①Yes ②No ③Don’t know

（2）Patients with cardiovascular diseases

①Yes ②No ③Don’t know

（3）People with low immunity

①Yes ②No ③Don’t know

13.Do you think the following are contraindications to pneumococcal vaccine (PPSV23) in the elderly?？

（1）Allergy to any ingredient in PPSV23

①Yes ②No ③Don’t know

（2）Suffering from a chronic disease but the current unstable condition control

①Yes ②No ③Don’t know

Part4．Pneumococcal disease and PPSV23 attitudes

14.What do you think about your risk of contracting pneumococcal infection？

□Very high □Fairly high □Normal □Low □Very low

15.What do you think about the risk of transmitting a pneumococcal infection to others?

□Very high □Fairly high □Normal □Low □Very low

16.How damaging do you think a pneumococcal infection is to your health?

□Very serious □Fairly serious □Normal □Not too serious □Not at all serious

17.How important do you think it is for your health to be vaccinated with PPSV23?

□Very important □Fairly important □Normal □Not too important □Completely unimportant

18.What do you think of the effectiveness of PPSV23？

□Very valid □Fairly valid □Normal □Not too valid □Completely invalid

19.What do you think about the safety of PPSV23？

□Very safety □Fairly safety □Normal □Not too safety □Very unsafe

20.Have you heard negative news about vaccines？

①Yes ②No

Part5．Influence of people around

21.In the past year, has anyone in your family/relatives/friends had a pneumococcal illness?

①Yes ②No

22.In the past year, has anyone in your family/relatives/friends vaccinated PPSV23？

①Yes ②No

23.Has a healthcare professional recommended PPSV23 to you in the past year？

①Yes ②No

Part6．Vaccination services

24.How much are you willing to vaccinate for a shot of PPSV23?

□ < 100yuan □100-200yuan □≥200yuan

Part7．Vaccination-related factors

25.How did you find out that you could vaccinate PPSV23? [Multiple choice question]

□Street (township) or community (village) publicity □Vaccination medical personnel publicity □Publicity by other medical personnel □Publicity by workplace □Informed by family members/relatives □Informed by friends/neighbors □Radio and TV □Internet publicity □Other [please specify].___________________________

26.What is the most important reason for you to vaccinate PPSV23？

（1）Protect myself with PPSV23 vaccination.

①Yes ②No

（2）Vaccination with PPSV23 to protect those around you.

①Yes ②No

（4）The new crown outbreak made me focus on preventing disease.

①Yes ②No

（4）There is a fee waiver for PPSV23 vaccination.

①Yes ②No

（5）I've been urged to get vaccinated.

①Yes ②No

（6）Other reasons [please specify]___________________________________

Unvaccinated Group Questionnaire:

Part1．Basic Information

1.Date of birth？（Not later than 30 September 1963）

□□□□year□□month□□date

2.Gender？

□Man □Women

3.Marital status？

□Married □Divorced/widowed

4.Educational level？

□Primary and below □Junior high school □Secondary/high school and above

5.Where have you lived most often in the past year？

□Countryside □Town

6.How many members of your family have lived together in the past year？（Note: Means living together for more than 6 months）

□1-2 □3-4 □≥5

7.What was your annual household income in the past year?(10,000 yuan) (Note: Refers to the total annual income of household members living together)

□0-4 □5-9 □≥10

Part2．Health status

8.What doctor-diagnosed chronic illnesses have you had in the past year？

□No □Yes

9.In the past year, have you experienced flu-like symptoms such as fever and coughing？

①No ②Yes

10.How do you feel about your health over the past year？

□Good □Fair □Poor

Part3．Pneumococcal diseases and PPSV23 related knowledge

11.Do you think the following statements are correct？

（1）Pneumococcus bacteria can be spread in the air when an infected person coughs or sneezes.

①Yes ②No ③Don’t know

（2）Pneumococcal infection can cause otitis media.

①Yes ②No ③Don’t know

（4）Pneumococcal infection can cause meningitis.

①Yes ②No ③Don’t know

（4）Vaccination can prevent pneumococcal diseases.

①Yes ②No ③Don’t know

（5）Fever may occur after PPSV23 vaccination.

①Yes ②No ③Don’t know

12. Do you consider the following to be priority populations for PPSV23 vaccination?？

（1）People over 60 years old

①Yes ②No ③Don’t know

（2）Patients with cardiovascular diseases

①Yes ②No ③Don’t know

（3）People with low immunity

①Yes ②No ③Don’t know

13.Do you think the following are contraindications to pneumococcal vaccine (PPSV23) in the elderly?？

（1）Allergy to any ingredient in PPSV23

①Yes ②No ③Don’t know

（2）Suffering from a chronic disease but the current unstable condition control

①Yes ②No ③Don’t know

Part4．Pneumococcal disease and PPSV23 attitudes

14.What do you think about your risk of contracting pneumococcal infection？

□Very high □Fairly high □Normal □Low □Very low

15.What do you think about the risk of transmitting a pneumococcal infection to others?

□Very high □Fairly high □Normal □Low □Very low

16.How damaging do you think a pneumococcal infection is to your health?

□Very serious □Fairly serious □Normal □Not too serious □Not at all serious

17.How important do you think it is for your health to be vaccinated with PPSV23?

□Very important □Fairly important □Normal □Not too important □Completely unimportant

18.What do you think of the effectiveness of PPSV23？

□Very valid □Fairly valid □Normal □Not too valid □Completely invalid

19.What do you think about the safety of PPSV23？

□Very safety □Fairly safety □Normal □Not too safety □Very unsafe

20.Have you heard negative news about vaccines？

①Yes ②No

Part5．Influence of people around

21.In the past year, has anyone in your family/relatives/friends had a pneumococcal illness?

①Yes ②No

22.In the past year, has anyone in your family/relatives/friends vaccinated PPSV23？

①Yes ②No

23.Has a healthcare professional recommended PPSV23 to you in the past year？

①Yes ②No

Part6．Vaccination service

24.How much are you willing to vaccinate for a shot of PPSV23?

□ < 100yuan □100-200yuan □≥200yuan

Part7．Factors associated with non-vaccination

25. What's the main reason you didn't vaccinate PPSV23?？

（1）I think the vaccine are too expensive.

①Yes ②No

（2）I have never heard of this disease and/or this vaccine.

①Yes ②No

（3）I don't know where I can vaccinate PPSV23.

①Yes ②No

（4）Places that can vaccinate PPSV23 are far from home.

①Yes ②No

（5）I'm in good health, I don't need vaccination.

①Yes ②No

（6）I'm waiting for a better pneumococcal vaccine to hit the market.

①Yes ②No

（7）I have contraindications/bad health.

①Yes ②No

（8）No one recommended this vaccine to me.

①Yes ②No

（9）Vaccine incidents in recent years have made me uneasy.

①Yes ②No

（10）Other reasons [please specify]___________________________________
